# Are scabies and impetigo “normalised”? A cross-sectional comparative study of hospitalised children in northern Australia assessing clinical recognition and treatment of skin infections

**DOI:** 10.1371/journal.pntd.0005726

**Published:** 2017-07-03

**Authors:** Daniel K. Yeoh, Aleisha Anderson, Gavin Cleland, Asha C. Bowen

**Affiliations:** 1 Department of Infectious Diseases, Princess Margaret Hospital for Children, Perth, Western Australia; 2 Division of Paediatrics, School of Medicine, University of Western Australia, Perth, Western Australia; 3 Paediatric Services, Western Australia Country Health Service Kimberley Region, Broome, Western Australia; 4 Paediatric Services, Hedland Health Campus, Port Hedland, Western Australia; 5 Wesfarmers Centre for Vaccines and Infectious Diseases, Telethon Kids Institute, University of Western Australia, Perth, Western Australia; 6 Menzies School of Health Research, Charles Darwin University, Darwin, Northern Territory; Hebrew University Hadassah Medical School, ISRAEL

## Abstract

**Background:**

Complications of scabies and impetigo such as glomerulonephritis and invasive bacterial infection in Australian Aboriginal children remain significant problems and the overall global burden of disease attributable to these skin infections remains high despite the availability of effective treatment. We hypothesised that one factor contributing to this high burden is that skin infection is under-recognised and hence under-treated, in settings where prevalence is high.

**Methods:**

We conducted a prospective, cross-sectional study to assess the burden of scabies, impetigo, tinea and pediculosis in children admitted to two regional Australian hospitals from October 2015 to January 2016. A retrospective chart review of patients admitted in November 2014 (mid-point of the prospective data collection in the preceding year) was performed. Prevalence of documented skin infection was compared in the prospective and retrospective population to assess clinician recognition and treatment of skin infections.

**Results:**

158 patients with median age 3.6 years, 74% Aboriginal, were prospectively recruited. 77 patient records were retrospectively reviewed. Scabies (8.2% vs 0.0%, OR N/A, p = 0.006) and impetigo (49.4% vs 19.5%, OR 4.0 (95% confidence interval [CI 2.1–7.7) were more prevalent in the prospective analysis. Skin examination was only documented in 45.5% of cases in the retrospective review. Patients in the prospective analysis were more likely to be prescribed specific treatment for skin infection compared with those in the retrospective review (31.6% vs 5.2%, OR 8.5 (95% CI 2.9–24.4).

**Conclusions:**

Scabies and impetigo infections are under-recognised and hence under-treated by clinicians. Improving the recognition and treatment of skin infections by clinicians is a priority to reduce the high burden of skin infection and subsequent sequelae in paediatric populations where scabies and impetigo are endemic.

## Author summary

Scabies and impetigo are common skin infections in children across the developing world as well as in disadvantaged populations living in developed countries. In previous studies including Australian Aboriginal and Pacific Islander children the rates of impetigo and scabies were amongst the highest described worldwide. The complications of these skin conditions include invasive bacterial infection, chronic kidney disease and potentially chronic heart disease and thus the burden of sequelae is significant. There are simple therapies which are efficacious in treating scabies and impetigo yet there has been little progress in reducing the burden of complications in endemic settings. We demonstrate in this study that scabies and impetigo, in a region with high prevalence of these conditions, are often not recognised by clinicians and appropriate treatment is not prescribed as a result of this. This is likely because clinicians ‘normalise’ skin infection where it is highly prevalent and therefore do not offer therapy unless specifically asked. The findings of this study have significant implication for health policy in regions with high prevalence of scabies and impetigo where focus should be put on improving health care worker recognition and awareness as well as exploring alternative strategies to individual case management of skin infection. Moreover future research should be directed at exploring the barriers to clinician recognition and treatment of these conditions and assessing specific strategies to ameliorate these.

## Introduction

Skin infections including scabies, impetigo, tinea and pediculosis are common in children, with high prevalence in developing countries and marginalised populations within developed countries[1–4]. It is estimated that 162 million children in low and low-middle income countries have active impetigo at any one time[1]. Similarly, scabies is very common in tropical environments particularly amongst children with population prevalence in excess of 10% in many Asian, Pacific Island and Central and South American countries[2]. Community based skin infection prevalence studies from Aboriginal populations in northern Australia demonstrate some of the highest prevalence rates of scabies and impetigo in the world[5, 6].

The high burden of skin infections is challenging in the primary health care setting, whilst the serious sequelae of skin infections predominantly affect hospitalised patients. The burden of these sequelae of skin infections is greatest where the prevalence is high[7–9]. Impetigo and secondarily infected scabies lesions may be complicated by invasive bacterial infections including cellulitis, skeletal infection and bacteraemia[10–13]. Immune complications of impetigo are important in tropical regions where *Streptococcus pyogenes* (Group A Streptococcus or GAS) is the predominant pathogen[4, 7, 14]. In endemic settings most cases of acute post streptococcal glomerulonephritis (APSGN) are preceded by impetigo[15] and scabies infestation has been shown to be associated with renal disease[16]. There is also a plausible link between *S*. *pyogenes* skin infection and acute rheumatic fever (ARF) and rheumatic heart disease[17].

There are effective and relatively well tolerated treatments for skin infections, yet the burden of disease appears to be increasing or at least stable in endemic settings[18–20]. Translation of these evidence-based treatments depends on the successful recognition and diagnosis of skin infection by clinicians.

We hypothesised that under-recognition due to the ‘normalisation’ of skin infection by clinicians contributes to under-treatment and the perpetuation of skin infection and subsequent sequelae in endemic settings. Normalisation is a term to describe that in contexts of high burden, but not life-threatening disease, clinicians may not specifically diagnose or treat scabies, impetigo, pediculosis or tinea when a patient presents to a health care provider for a reason other than skin infection because these infections are so common they are regarded as normal and are thus ignored or forgotten. The term normalisation has been used previously in the Australian[21–23] and international[24] literature to describe this hypothesized phenomenon in regions with endemic skin infection. Clinician under-recognition of scabies was suggested in the findings of one previous study in a population with high prevalence of scabies[25] but thus far normalisation of impetigo has not been demonstrated in the clinical setting. To test our hypothesis, we designed this study to prospectively assess prevalence of skin infection and to assess recognition by comparing this with the documented prevalence in a retrospective case note review.

## Methods

### Ethics statement

Participation in the study was voluntary and verbal and written informed consent was sought from each participant’s parent or appropriate guardian and where appropriate, (i.e. in children >7yo) assent was obtained. Ethics approval was granted by the Western Australian Country Health Service Research Ethics Committee (project number 2015:11) and the Western Australian Aboriginal Health Ethics Committee (project number 635). The study protocol was finalised only after consultation with the Kimberley Aboriginal Health Planning Forum Environmental Health and Research subcommittees as well as local Aboriginal Medical Services.

### Study design

We performed a prospective, cross-sectional study to ascertain prevalence of skin infection and compared this with a retrospective, cross-sectional study to assess recognition of skin infection by health professionals. In the prospective arm, patients were opportunistically recruited from two regional hospitals during the period from October 2015 to January 2016. Data for the retrospective review were obtained from the medical records of all patients admitted to the two paediatric wards in November 2014 at both centers. The retrospective data collection was limited to a one-month period that was the mid-point in the prospective data collection period for feasibility. This retrospective data capture provided the same season and likely admission profile as the prospective study.

### Study population and setting

All children and adolescents (aged <16 years) admitted to Broome Hospital (a 36 bed facility with 8 paediatric inpatient beds) and Hedland Health Campus (a 55 bed facility with 8 paediatric inpatient beds) during the study period were eligible for participation in the prospective study. These two hospitals provide the regional paediatric services for the Kimberley and Pilbara regions respectively, covering a total catchment of greater than 900 000 km^2^ in the north of the state of Western Australia. In combination, these units admit around 1500 paediatric patients annually[26] servicing a total paediatric population of over 20 000 [27]. The study was conducted during the tropical “wet season” when temperatures are on average 33–36^°^C respectively and rainfall and humidity are high[27]. The population of the Kimberley region is 37,000 of whom 40% are Aboriginal[27] The population of the Pilbara is 62,000 of whom 12% are Aboriginal[27].

Patients were recruited by the site coordinator (DY, AA) at the two sites. An attempt to approach all admitted patients was made by the respective site coordinators and individuals were approached to participate regardless of the reason for admission, co-morbidities, ethnicity, language spoken, address or gender. Individuals were excluded if the individual or the parent / carer did not assent or consent to participation or if there was no parent / carer available to provide consent.

### Data collection and management

All participants in the prospective arm of the project underwent assessment including:

1. directed history including age, ethnicity, locality of residence, number of household members, primary reason for admission, past history of skin infection, treatment and complications
2. a full examination of the skin looking specifically for scabies (including secondarily infected lesions), impetigo (flat/dry, crusted or purulent lesions), tinea corporis, tinea capitis and pediculosis.
3. any treatment prescribed for skin infection was recorded and classified as either primarily for skin infection or for another indication but also covering skin infection (e.g. osteomyelitis, septic arthritis as the primary condition under treatment).

All purulent or crusted skin lesions were swabbed and sent for microscopy, culture and antibiotic susceptibility testing according to standardized methods. All samples were delivered to the local microbiology laboratory and subsequently sent to a tertiary laboratory (over 2500km away) for processing.

In the retrospective review of medical records age, ethnicity, locality of residence, number of household members, primary reason for admission, history of skin infection (including complications and comorbidities), documentation of skin infection on physical examination and treatment recommendations were recorded. Pathology records were reviewed for each patient and microbiology of skin lesions was recorded where applicable.

All data were initially recorded onto standard case report forms and subsequently entered onto a secure online database (REDCap Software–Version 6.10.12).

### Definitions

Ethnicity was self-reported or as recorded in the medical record as Aboriginal and/or Torres Strait Islander (ATSI), Pacific Islander / Maori, Caucasian or other. Locality was assessed and classified for each participant as a local town (resident in Broome or Port Hedland), another regional town (within the region but outside of Broome and Port Hedland) or a remote Aboriginal community. By the Australian Standard Geographical Classification (ASGC) for remoteness, Broome, Port Hedland and Karratha are considered remote and the remainder of the Kimberley and Pilbara are considered very remote[28]. Remote Aboriginal communities are defined areas inhabited by Aboriginal people with housing and infrastructure that is managed on a community basis[29]. Whilst nearly all are connected to essential services, disruptions are commonplace with 38%, 85% and 49% of communities reporting disruptions to water supply, electricity and sewage disposal respectively in 2001[29]. Access to health services is often limited with 74% of communities 100km or further from the nearest hospital and only 12% with a local doctor resident in the community[29].

Skin infection was defined as scabies, impetigo, tinea corporis, tinea capitis and/or pediculosis. A standard diagnostic guideline with clinical and photographic definitions of each condition was used as a reference tool in the prospective assessment of skin infection[30]. Scabies, a parasitic infection of the skin was diagnosed in the presence of pruritic papules in a typical distribution; classically in the web spaces, hands, feet and other moist areas. Impetigo is a bacterial infection of the superficial skin. Clinical detection ranges from active purulent or crusted lesions to resolving flat, dry lesions. Tinea infections were diagnosed based on well-demarcated areas of scale with a raised edge and itch. Pediculosis was diagnosed if either the live lice or nits attached to strands of hair were visualized. Secondary bacterial infection of parasitic or fungal lesions was diagnosed in the presence of pus or a crust. Cellulitis and abscess were not included in this assessment. Each condition was diagnosed clinically by the site’s study coordinator (a paediatric clinician) and treatment was prescribed according to local guidelines[31].

### Statistical analysis

The primary objective was to compare the prevalence of skin infection in the prospective study with the documented prevalence in the retrospective review. Secondary objectives were to compare the frequency of treatment prescribed for skin infections between the prospective and the retrospective groups, to assess the local microbiology of impetigo in the prospective group and to assess age, admission reason, remoteness, overcrowding and ethnicity as potential risk factors for skin infection in the prospective study. The demographic details of patients and microbiology of impetigo lesions were reported for each group using descriptive statistics. Prevalence of each of the skin infections (scabies, impetigo, tinea, and pediculosis) in the prospective and retrospective cohort was determined. Odds ratios comparing frequency of each skin infection and frequency of treatment prescribed in the prospective and retrospective cohorts were calculated using logistic regression. Univariate analysis of associations between skin infection and age, admission reason, remoteness, overcrowding and ethnicity respectively was performed using logistic regression. Comparison of categorical data was conducted using logistic regression, Pearson’s Chi-square test or Fisher’s exact test (where 0 events occurred in one or more groups). P-values of <0.05 were considered to indicate statistical significance. All statistical analysis was performed using SPSS statistics version 23.0.0.0 (Armonk, NY: IBM Corp.).

## Results

### Study population

**Fig 1:**
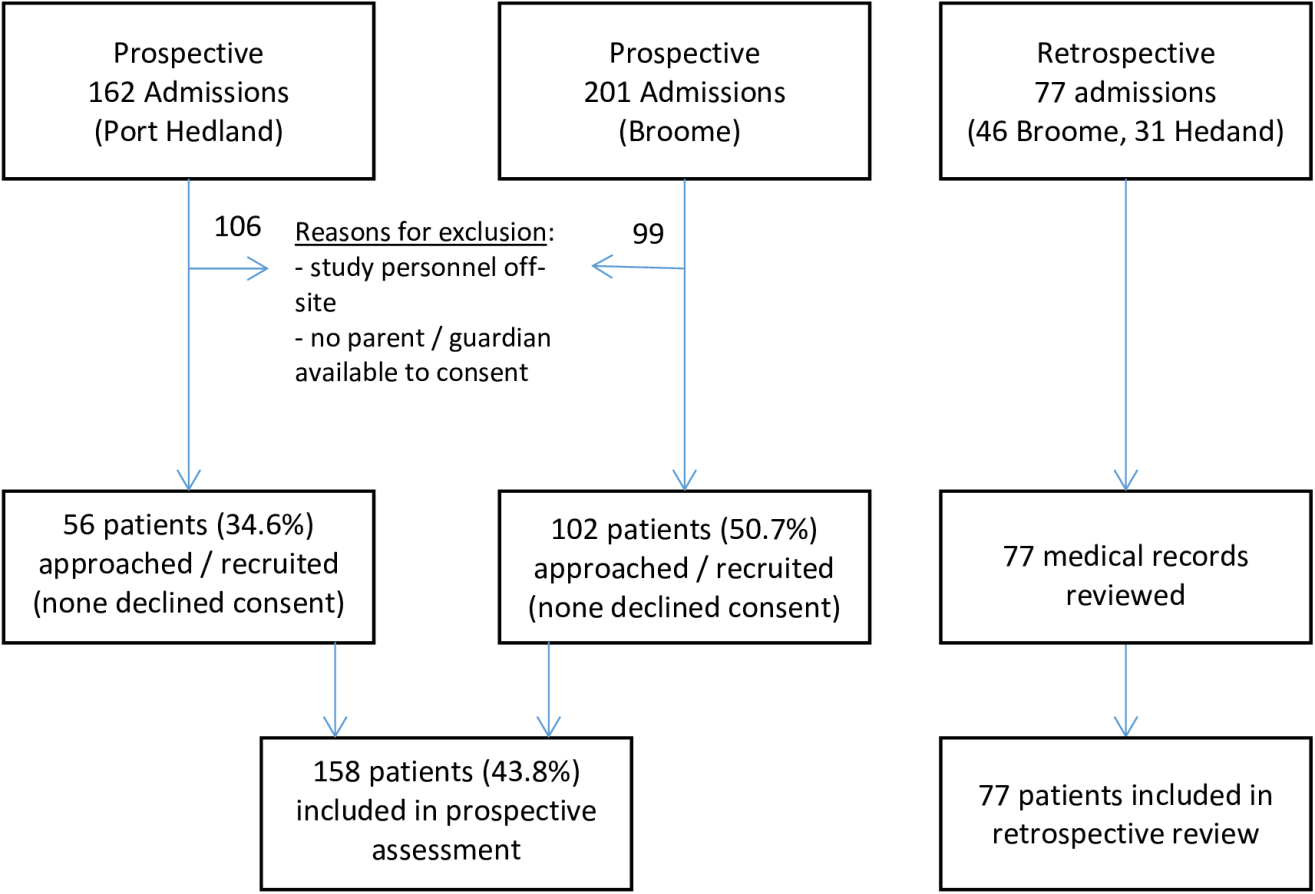
Study flowchart.

One hundred and fifty-eight patients were included in the prospective assessment; 102 from Broome and 56 from Port Hedland. This was 43.8% of those admitted during the period (Fig 1). No patients who were approached to participate refused to consent. Seventy-seven patient records were reviewed in the retrospective arm; 46 from Broome and 31 from Port Hedland.

The demographic characteristics including age, gender, ethnicity and area of residence along with the primary reason for admission were similar between the prospective and retrospective cohorts (Table 1). In the prospectively assessed group median age was 3.6 years (interquartile range [IQR] 0.9–7.4), 74.1% were of Aboriginal ethnicity and 25.3% were from a remote Aboriginal community. The most common reason for admission was respiratory illness (34.2% prospective, 28.6% retrospective). Conditions that may complicate skin infection including APSGN, ARF, skeletal infections and soft-tissue infections accounted for almost one-quarter of admissions (24.1% prospective, 20.8% retrospective).

**Table 1:** Baseline characteristics–prospective vs retrospective.

Characteristic	Prospective Cases (n = 158)	Retrospective Review (n = 77)	P-value*
**Hospital:**			
Broome n (%)	102 (64.6%)	46 (59.7%)	0.473
Port Hedland n (%)	56 (35.4%)	31 (40.3%)	
**Age in years**: median (IQR)	3.6 (0.9, 7.4)	4.1 (1.4, 9.5)	0.746^#^
**Gender:** Male, n (%)	86 (54.4%)	43 (55.8%)	0.838
**Ethnicity:**			
Aboriginal / Torres Strait Islander (ATSI), n (%)	117 (74.1%)^	50 (64.9%)	0.148
**Community of residence:**			
local town, n (%)	92 (58.2%)	52 (67.5%)	0.374
other town, n (%)	26 (16.5%)	9 (11.7%)	
remote community, n (%)	40 (25.3%)	16 (20.8%)	
**Primary Admission Reason:**			
APSGN, n (%)	7 (4.4%)	4 (5.2%)	0.741
ARF, n (%)	4 (2.5%)	2 (2.6%)	
bone / joint infection, n (%)	3 (1.9%)	1 (1.3%)	
SSTI, n (%)	24 (15.2%)	9 (11.7%)	
Respiratory, n (%)	54 (34.2%)	22 (28.6%)	
Gastroenteritis, n (%)	23 (14.6%)	13 (16.9%)	
UTI/CNS, other infection, n (%)	8 (5.1%)	2 (2.6%)	
Injury / Immersion, n (%)	9 (5.7%)	6 (7.8%)	
Surgical / ENT / Dental, n (%)	13 (8.2%)	8 (10.4%)	
FTT, n (%)	3 (1.9%)	0 (0.0%)	
Other, n (%)	10 (6.3%)	10 (13.0%)	

*Pearson’s Chi-square (unless otherwise specified)

# Mann Whitney U Test

^compared with overall ATSI population 12% in Pilbara and 40% in Kimberley in 2011 (22)

APSGN: Acute post streptococcal glomerulonephritis, ARF: acute rheumatic fever; SSTI: skin and soft tissue infection, ENT: ear nose or throat infection, UTI: urinary tract infection, CNS: central nervous system infection, FTT: failure to thrive

Less than 20% of patients in the prospective analysis reported receiving specific treatment for skin infection in the twelve months prior to admission; 13.9% of patients had received treatment for scabies, 18.4% for impetigo and 13.9% for pediculosis.

### Prevalence, recognition and treatment of skin infection

Prevalence of skin infection was high in the prospectively assessed group with 53.2% of patients diagnosed with one or more skin infections. Scabies was diagnosed in 8.2% (95% confidence interval [CI] 3.9–12.6). Impetigo was present in 49.4% (95%CI 41.5–57.3%) of participants, with crusted or purulent lesions identified in 27.8% (95% CI 20.8–34.9%)tinea was identified in in 8.2% (95% CI 3.9–12.6%) and pediculosis in 14.6% (95% CI 9.0–20.1%).

The recognition of skin infection was four-fold greater in the prospective assessment compared with the retrospective review; odds ratio (OR) 4.0 (95% CI 2.2–7.5) (Table 2). Skin infection was diagnosed in 22.1% of patients in the retrospective review and notably 54.5% did not have any skin examination findings documented anywhere in the case notes. The prevalence of scabies (8.2% vs 0.0%; OR N/A (p = 0.006)), impetigo (49.4% vs 19.5%; OR 4.0 (2.1–7.7)) and pediculosis (14.6% vs 1.3%; OR 13.0 (1.7–97.8)) respectively were significantly higher in the prospective assessment compared with the retrospective review. Prevalence of tinea was not significantly higher in the prospective assessment (8.2% vs 2.6%; OR 3.4 (0.7–15.3))

**Table 2:** Prevalence of skin infection–prospective vs retrospective.

Skin Infection	Prospective Cases (n = 156)	Retrospective Review (n = 77)	OR (95% CI)	p-value
**Any skin lesions:** Total n (%)	84 (53.2%)	17 (22.1%)	**4.0 (2.2–7.5)**	**<0.001**
**Scabies:** Total n (%)	13 (8.2%)	0 (0%)	**N/A**	**0.006**^#^
**Impetigo (total)**: Total n (%)	78 (49.4%)	15 (19.5%)	**4.0 (2.1–7.7)**	**<0.001**
**Impetigo (purulent/crusted):** Total n (%)	44 (27.8%)	12 (15.6%)	**2.1 (1.0–4.2)**	**0.041**
**Tinea Corporis / Capitis:** Total n (%)	13 (8.2%)	2 (2.6%)	3.4 (0.7–15.3)	0.117
**Pediculosis:** Total n (%)	23 (14.6%)	1 (1.3%)	**13.0 (1.7–97.8)**	**0.013**

^#^Fisher’s exact test (2-sided) used where 0 events in retrospective arm

Specific treatments for scabies, impetigo, tinea and/or pediculosis were prescribed eight times more frequently in the prospective assessment (31.6% vs 5.2%; OR 8.5 (95% CI 2.9–24.4)) (Table 3). Antibiotics were the most commonly prescribed treatment and significantly more patients in the prospective analysis received antibiotics for skin infection (27.8% vs 14.3%; OR 2.3 (1.1–4.8)). No patients received treatment for scabies or tinea in the retrospective group while all patients diagnosed in the prospective assessment received treatment. Environmental health measures were implemented in 8.2% of the prospective cases compared with none in the retrospective review (p = 0.006). Although skin infection recognition was higher in the prospective arm, the communication of this finding was poor. In a subsequent review, only 64.6% (31 of 48) of children who received treatment for skin infection in Broome in the prospective study had this documented in the discharge letter.

**Table 3:** Treatment of skin infection prospective vs retrospective.

Treatment	Prospective Cases (n = 156)	Retrospective Review (n = 77)	OR (95% CI)	p-value
**Treatment recommended (specifically for skin)**^#^**:** Total n (%)	50 (31.6%)	4 (5.2%)	**8.5 (2.9–24.4)**	**<0.001**
**Treatment recommended (any covering skin infection)**^**:** Total n (%)	62 (39.2%)	12 (15.6%)	**3.5 (1.8–7.0)**	**<0.001**
**Treatment Type:**				
IV antibiotics n (%)	21 (13.3%)	9 (11.7%)	1.2 (0.5–2.7)	0.730
IM antibiotics n (%)	6 (3.8%)	1 (1.3%)	3.0 (0.4–25.4)	0.313
Oral antibiotics n (%)	24 (15.2%)	6 (7.8%)	2.1 (0.8–5.4)	0.117
Any antibiotic n (%)	44 (27.8%)	11 (14.3%)	**2.3 (1.1–4.8)**	**0.024**
Oral scabicide n (%)	1 (0.6%)	0 (0%)	N/A	1.000
Topical scabicide n (%)	14 (8.9%)	0 (0%)	**N/A**	**0.006**
Oral antifungal n (%)	4 (2.5%)	0 (0%)	N/A	0.306
Topical antifungal n (%)	10 (6.3%)	0 (0%)	**N/A**	**0.033**
Pediculosis treatment n (%)	22 (13.9%)	2 (2.6%)	**6.1 (1.4–26.5)**	**0.017**
Environmental Health measures^**^: recommended n (%)	13 (8.2%)	0 (0%)	**N/A**	**0.006**

# treatment specifically for skin infection (not otherwise indicated)

^treatment for skin infection specifically or for another indication but also treating skin infection (eg anti-staphylococcal antibiotics for osteomyelitis)

^**^environmental health measures–treatment of household contacts and education around cleaning household linen and clothing

IV = intravenous, IM = intramuscular

### Risk factors and associations

Household overcrowding, Aboriginal ethnicity, older age (>5yo) and residence in a remote community were all associated with an increased odds of skin infection in the prospectively assessed group (Table 4). Scabies, impetigo and pediculosis were all significantly associated with Aboriginal ethnicity and household overcrowding. Increased age was associated with increased prevalence of impetigo and pediculosis whilst prevalence of scabies and tinea was similar between age groups (Table 5). Children aged >5y were 3 times more likely to have a condition complicating skin infection (APSGN, ARF, bone and joint infection and soft tissue infection) as the primary admission diagnosis compared with those <5y age; OR 3.2 (95%CI 1.5–6.9).

**Table 4:** Risk factors for skin infection (prospective only–univariate logistic analysis).

Characteristic	Any lesions: OR (95% CI)	Scabies: OR(95% CI)	Impetigo: OR(95% CI)	Tinea: OR(95% CI)	Pediculosis: OR (95% CI)
**Ethnicity**:					
- Non-ATSI	1 (ref)	1 (ref)	1 (ref)	1 (ref)	1 (ref)
- ATSI	**28.5 (8.3–98.4)**	N/A * p = 0.022	**36.2 (8.3–157.4)**	4.6 (0.6–36.3)	N/A ** p = 0.001
**Remoteness**:					
- Town	1 (ref)	1 (ref)	1 (ref)	1 (ref)	1 (ref)
- Remote community	**7.7 (3.0–19.8)**	1.4 (0.4–4.6)	**9.5 (3.7–24.5)**	2.8 (0.9–8.9)	2.2 (0.9–5.5)
**Household**:					
<5 members	1 (ref)	1 (ref)	1 (ref)	1 (ref)	1 (ref)
>5 members	**3.9 (1.9–7.9)**	**4.0 (1.1–14.0)**	**3.4 (1.7–6.8)**	2.2 (0.7–7.0)	**4.2 (1.7–10.8)**

* no cases of scabies in non-Aboriginal patients (p-value 0.022) (Fisher’s)

** no cases of pediculosis in non-Aboriginal patients (p-value 0.001) (Fisher’s)

ATSI—Aboriginal and Torres Strait Islander

**Table 5:** Prevalence by age group (prospective).

Age (n)	<1mo (6)	1-5mo (18)	6-12mo (18)	1-4yo (52)	5-10yo (43)	>10yo (21)	p-value^
**Any skin lesions:** prevalence	0%	33.3%	33.3%	55.8%	62.8%	76.2%	**0.002**
**Scabies:** prevalence	0%	11.1%	11.1%	5.8%	9.3%	9.5%	0.917
**Impetigo (total):** prevalence	0%	27.8%	33.3%	48.1%	62.8%	71.4%	**0.003**
**Tinea:** prevalence	0%	5.6%	5.6%	9.6%	9.3%	9.5%	0.952
**Pediculosis:** prevalence	0%	0%	5.6%	9.6%	32.6%	14.3%	**0.004**

^ Pearson’s chi-square test

Where the reason for admission was a condition associated with or complicating skin infection the prevalence of impetigo was significantly higher compared with admission for other reasons (Table 6). Despite this difference, skin infection was still common amongst those admitted for reasons not directly associated with or complicating skin infection with 40.5% (95% CI 31.6–49.4%) of these patients having any skin infection: scabies in 6.6% (95% CI 2.1–11.1%), impetigo in 35.5% (95% CI 26.9–44.2%), crusted/purulent impetigo in 13.2% (95% CI 7.1–19.4%), tinea capitis / corporis in 5.8% (95% CI 1.6–10.0%) and pediculosis in 9.9% (95% CI 4.5–15.3%).

**Table 6:** Prevalence by admission reason (prospective).

Admission reason	SSTI / BJI / ARF / APSGN (37)	Other (121)	OR (95% CI)	p-value
**Any skin infections:** prevalence	35 (94.6%)	49 (40.5%)	25.7 (5.9–111.9)	**<0.001**
**Scabies:** prevalence	5 (13.5%)	8 (6.6%)	2.2 (0.7–7.2)	0.190
**Impetigo (total)**: prevalence	35 (94.6%)	43 (35.5%)	31.7 (7.3–138.5)	**<0.001**
**Tinea:** prevalence	6 (16.2%)	7 (5.8%)	3.15 (1.0–10.1)	0.052
**Pediculosis:** prevalence	11 (29.7%)	12 (9.9%)	3.8 (1.5–9.7)	**0.004**

### Microbiology of skin infections

**Fig 2:**
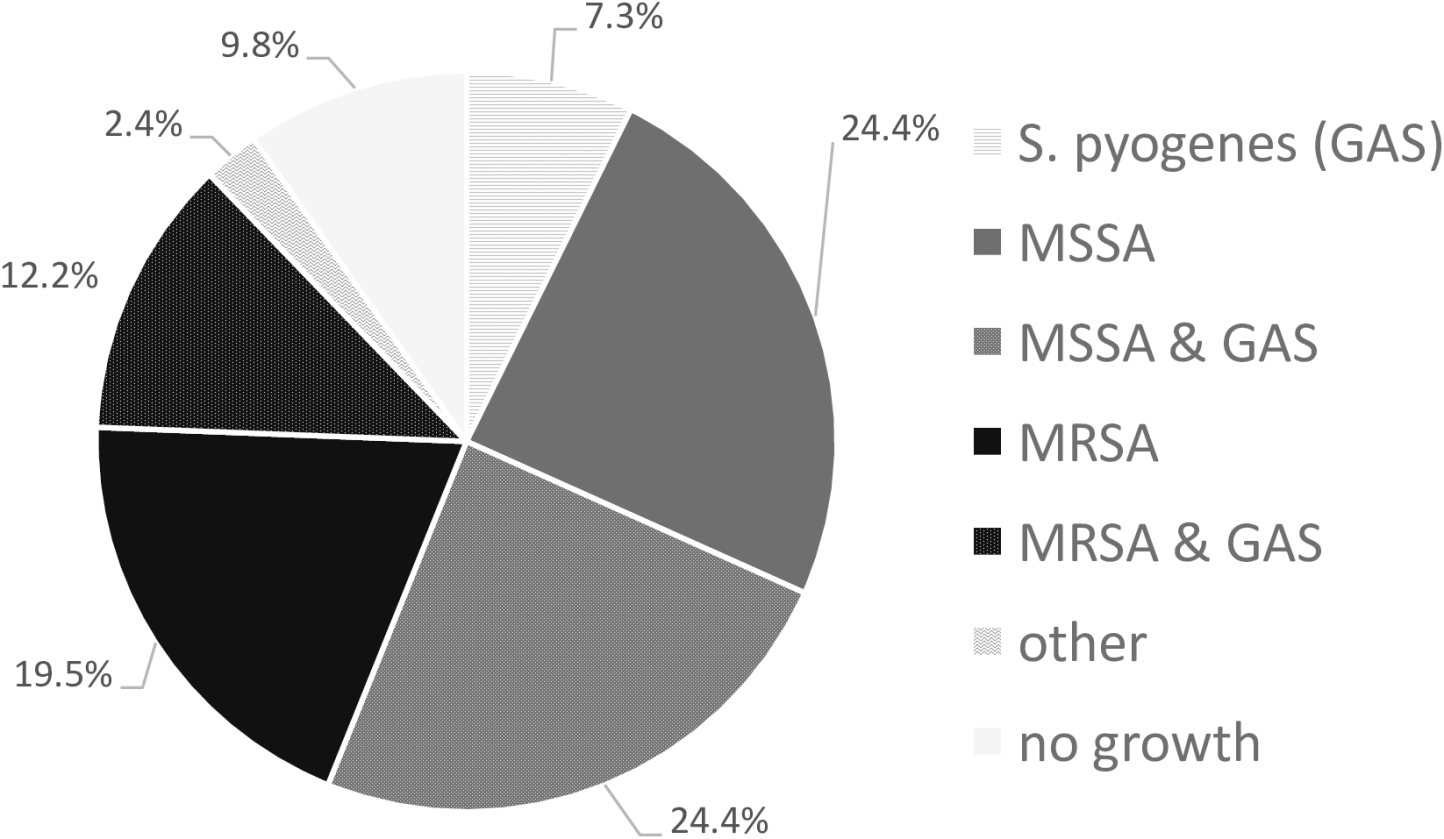
Impetigo microbiology. The figure represents a total of 41 culture positive microbiological samples from prospectively assessed patients with crusted or purulent impetigo.

Bacterial swabs were collected from 41/ 44 (93.2%) participants with crusted and purulent impetigo lesions in the prospective assessment. An organism was isolated in 37/41 (91%). *Staphylococcus aureus* (30/37, 81% of swabs) and *Streptococcus pyogenes* (18/41, 44%) were the predominant pathogens and co-infection (15/41, 37%) was common (Fig 2). Of the *S*. *aureus* isolates 13 (39%) were methicillin resistant (MRSA). All *S*. *aureus* isolates were susceptible to co-trimoxazole (SXT). 90% of methicillin susceptible *S*. *aureus* (MSSA) and 77% of MRSA isolates were susceptible to clindamycin. All *S*. *pyogenes* were susceptible to penicillin. Susceptibility of *S*. *pyogenes* to SXT is not routinely tested in our laboratory.

## Discussion

Our findings demonstrate that under-recognition of skin infections is clearly an important problem; consequentially specific treatment for skin infections is not offered by clinicians. Previous studies have confirmed that under-recognition of scabies occurs in industrialised [32] and resource limited settings [25] due to the absence of diagnostic tests. In a cross-sectional study of a population dwelling in a slum in Brazil, Heukelbach *et al* found that despite an estimated community prevalence of scabies of 8.8%, doctors at the health care centre failed to diagnose any cases of scabies amongst the 260 patients who presented for other reasons during the study period[25]. Hong *et al* demonstrated that time constraints and Emergency Department (ED) overcrowding were potential factors contributing to the missed diagnosis of scabies in patients admitted to a tertiary hospital in Taiwan where the diagnosis was missed by the ED physician in 65% (72 of 111) of cases[32]. Although under-recognition of scabies has been previously described, this is the first comparative study to demonstrate the under-recognition by clinicians of impetigo along with scabies and pediculosis in a region of high skin infection prevalence. It is plausible that under-recognition and normalisation of skin infection contribute to the ongoing burden of skin infection in many endemic settings around the world and the findings of this study have significant implications in the context of the global burden of skin infections.

The implications of under-recognition of skin infection are manifold. The significant individual complications of scabies, pediculosis and impetigo are well documented[3, 4, 7–9, 17, 18]. Scabies, impetigo and pediculosis are highly transmissible and the assessment and treatment of household members is recommended as important public health control measures to prevent onward transmission and re-infection[33, 34]. In failing to treat children and their family members for skin infections during a hospital admission, clinicians may miss the opportunity to prevent serious individual complications, perpetuate the cycle of ongoing transmission within communities and possibly render other patients at risk by failing to implement appropriate infection control measures.

The prevalence of skin infection is high in this region as demonstrated in our prospective assessment with over half of all children having at least one form of skin infection. Limited data exists regarding prevalence of skin infection in the Kimberley and Pilbara region although other studies in regional Western and Northern Australia have found similarly high rates[5, 6, 35]. This high prevalence in our study supports our hypothesis that the under-recognition of skin infection is not a product of lack of familiarity with skin infection but rather that clinicians may ‘normalise’ skin infection because it is pervasive.

*S*. *aureus* was the most common pathogen in impetigo lesions in our study with *S*. *pyogenes* implicated in less than half of cases. In previous studies *S*. *pyogenes* has been demonstrated as the predominant pathogen in impetigo in tropical regions where co-infection with *S*. *aureus* is also common[14]. The yield of *S*. *pyogenes* culture may have been reduced in our study due to the processing of samples which were sent to a tertiary laboratory over 2500km away for plating. *S*. *pyogenes* requires rigorous temperature and humidity controls to prevent deterioration[36]. Our results also reflect the increasing importance of community acquired MRSA as a pathogen in the Australian setting with almost 50% of *S*. *aureus* demonstrating methicillin resistance, as has been apparent in recent times[11].

Clearly other broad factors including the social determinants of health and patient access to health services significantly contribute to the persistent high burden of skin infection and complications in this setting. Overcrowding, poor access to water and poverty have been associated with scabies and impetigo[1, 2, 9, 37], and indeed in our prospective analysis those patients from overcrowded households and remote Aboriginal communities had significantly increased odds of skin infection. As evidenced in the industrialisation of tropical countries such as Singapore, improvements in housing along with better access to quality healthcare can significantly impact on the burden of skin infection complications such as post-streptococcal glomerulonephritis[38].

It is reasonable to conceive that skin infection is also under-recognised outside of the hospital setting. This is compounded by the poor communication of diagnosis and treatment of skin infections documented by hospital staff on discharge back to primary care providers. Despite the high prevalence of skin infection in our population, few children had received specific treatment for impetigo in the preceding twelve months. This speaks to the likely ‘normalisation’ of skin sores amongst patients and families as well as under-treatment of skin infection in primary health care and community clinics. Strategies to improve recognition of and awareness of the complications of skin infection and the importance of treatment should consider primary health care workers, community workers, teachers, environmental health providers as well as children and their families.

This study has several limitations. Firstly, we were not able to recruit all of the eligible patients during the study period due to the availability of study staff and clinical commitments at other sites. Although recruitment was opportunistic, all children admitted to the ward were approached to participate when the study site doctor was present in order to achieve a representative sample. Secondly, in the prospective assessment, the diagnosis of skin infection was made clinically. In order to limit possible bias due to the reliance on clinical judgement, a diagnostic guide with clear clinical definitions was used as a reference tool in all cases[30]. Thirdly, although there were no major changes to public health policy in the region during the study period, the period encompassing the retrospective and prospective data collection was concurrent with an ongoing APSGN outbreak in the Kimberley region dating from the end of 2013[39] with an ongoing public health response from mid-2014 including the formation of the Kimberley Skin Health Regional Partnership in September 2014[40] and the revision of the Kimberley Aboriginal Medical Services Council’s skin infection treatment protocol in December 2014[31]. The heightened awareness of the importance of skin health at a regional level during late 2014, as evident in the public health response, could potentially have increased the recognition of skin infection during the retrospective data collection period and the coordinated public health response led to a documented decrease in the community prevalence of scabies between December 2014 and May 2015[41]. Despite these potential influences our study still found that skin infection was less likely to be diagnosed and treated in the retrospective period (November 2014) compared with the prospective data collection period. Finally, by virtue of the study design the assessment of clinician recognition of skin infection was performed retrospectively. Although it is plausible that clinicians did in fact recognise skin infection but did not document this, specific treatment for skin infection was prescribed far less frequently in the retrospective analysis supporting the finding of clinician under-recognition. Notwithstanding the limitations of this study the findings have significant implications for policy and future research.

As the diagnosis of skin infections in endemic settings remains predominantly clinical, the training of health care providers is vital in improving recognition. Strategies which potentially overcome the difficulties associated with the diagnosis and treatment of individual patients such as community dermatology[42–44] and mass drug administration[45] should be considered. Specific training of health workers has been shown to improve recognition and treatment of skin infection in resource-poor settings[46]. The use of integrated algorithms for the management of skin infection in health clinics has also demonstrated promise as a strategy to improve diagnosis of skin infection[47, 48]. Improvements in the accessibility to and availability of appropriate existing diagnostic tools such as dermatoscopy in resource limited settings should occur in parallel with further research exploring novel, practical diagnostic techniques[49, 50]. Community dermatology has demonstrated promise as an effective and affordable strategy in addressing common skin conditions including infections in low resource settings by addressing skin disease at a community level[42–44]. This approach encompasses several strategies including the training of community health care workers to recognize and treat skin disease, public health measures to address the determinants of skin disease, the education of community members and the prioritization of conditions to tackle based on accurate epidemiological data and simplifying their treatment[51]. Furthermore, the use of mass drug administration to target scabies has shown promise in populations with endemic disease; this strategy may allow circumvention of some of the challenges around clinical under-recognition and normalization of skin infection in selected settings[45].

Other factors outside of lack of skills and training likely contribute to the under-recognition of skin infection and warrant further investigation and intervention. Identifying barriers to clinician diagnosis and treatment of skin infection and exploring reasons for the ‘normalisation’ of skin infection through well-designed qualitative research is vital. Measuring potential under-recognition of skin infection at a patient and community level and exploring factors that contribute should be a focus of future studies. Certainly the phenomenon of normalisation has been described at the community level[21, 24] and may present a major barrier to health seeking and access to appropriate treatment in resource limited settings with endemic disease[25, 52]. It follows that any strategies to improve recognition and treatment of skin infection must consider and partner with the members of the communities which are affected[21]. Moreover, ongoing efforts to address the social determinants which lead to the disproportionate load borne by people of Aboriginal ethnicity, particularly those living in remote communities, remain of great importance[8, 21]. On a broader scale, skin infections are neglected at a global level with regard to prioritizing funding and policy development despite causing significant morbidity and mortality in resource-limited settings[53]. Our findings of under-recognition and under-treatment of skin infection in the clinical setting highlight some of the difficulties with addressing these conditions in disadvantaged populations and affirms the ongoing need for advocacy and a coordinated global approach to tackle common skin infections such as scabies and impetigo[1, 9].

## Conclusion

Skin infections are under-recognised by clinicians and this leads to suboptimal treatment and likely contributes to the significant ongoing burden of sequelae. There are many factors which contribute to the challenge of addressing the problem of skin infections and improving clinician recognition and treatment of skin infections is a priority.

## Supporting information

S1 Strobe Checklist (DOC)
