# Supplementary material for: Are scabies and impetigo “normalised”? A cross-sectional comparative study of hospitalised children in northern Australia assessing clinical recognition and treatment of skin infections
Source: PLoS Negl Trop Dis. 2017 Jul 3;11(7):e0005726. doi: 10.1371/journal.pntd.0005726 (PMC5510902; doi:10.1371/journal.pntd.0005726)
Supplement: S1 Strobe Checklist — (DOC) [file pntd.0005726.s001.doc]

STROBE Statement—Checklist of items that should be included in reports of ***cross-sectional studies***

|  | Item No | Recommendation |
| --- | --- | --- |
| **Title and abstract** | 1 | (*a*) Indicate the study’s design with a commonly used term in the title or the abstract  **[line 1]** |
|  |  | (*b*) Provide in the abstract an informative and balanced summary of what was done and what was found **[line 34]** |
| Introduction | | |
| Background/rationale | 2 | Explain the scientific background and rationale for the investigation being reported **[line 90-131]** |
| Objectives | 3 | State specific objectives, including any prespecified hypotheses **[line 222-227 and line 118-120]** |
| Methods | | |
| Study design | 4 | Present key elements of study design early in the paper [**line 133 to 142]** |
| Setting | 5 | Describe the setting, locations, and relevant dates, including periods of recruitment, exposure, follow-up, and data collection **[line 144 to 189]** |
| Participants | 6 | (*a*) Give the eligibility criteria, and the sources and methods of selection of participants [**line 145 to 148]** |
| Variables | 7 | Clearly define all outcomes, exposures, predictors, potential confounders, and effect modifiers. Give diagnostic criteria, if applicable **[line 191 to 230]** |
| Data sources/ measurement | 8* | For each variable of interest, give sources of data and details of methods of assessment (measurement). Describe comparability of assessment methods if there is more than one group [**line 164 to 219]** |
| Bias | 9 | Describe any efforts to address potential sources of bias **[line 157 to 162]** |
| Study size | 10 | Explain how the study size was arrived at **[line 134 to 137 and line 139 to 144]** |
| Quantitative variables | 11 | Explain how quantitative variables were handled in the analyses. If applicable, describe which groupings were chosen and why **[N/A]** |
| Statistical methods | 12 | (*a*) Describe all statistical methods, including those used to control for confounding **[line 221 to 238]** |
|  |  | (*b*) Describe any methods used to examine subgroups and interactions**[N/A]** |
|  |  | (*c*) Explain how missing data were addressed **[N/A]** |
|  |  | (*d*) If applicable, describe analytical methods taking account of sampling strategy **[N/A]** |
|  |  | (*e*) Describe any sensitivity analyses **[N/A]** |
| Results | | |
| Participants | 13* | (a) Report numbers of individuals at each stage of study—eg numbers potentially eligible, examined for eligibility, confirmed eligible, included in the study, completing follow-up, and analysed [**line 252 to 256]** |
|  |  | (b) Give reasons for non-participation at each stage **[line 258]** |
|  |  | (c) Consider use of a flow diagram **[line 258]** |
| Descriptive data | 14* | (a) Give characteristics of study participants (eg demographic, clinical, social) and information on exposures and potential confounders **[line 261 to 278]** |
|  |  | (b) Indicate number of participants with missing data for each variable of interest **[N/A]** |
| Outcome data | 15* | Report numbers of outcome events or summary measures **[line 285 to 332]** |
| Main results | 16 | (*a*) Give unadjusted estimates and, if applicable, confounder-adjusted estimates and their precision (eg, 95% confidence interval). Make clear which confounders were adjusted for and why they were included **[line 303, line 322, line 344, line 354]** |
|  |  | (*b*) Report category boundaries when continuous variables were categorized [**N/A]** |
|  |  | (*c*) If relevant, consider translating estimates of relative risk into absolute risk for a meaningful time period [**N/A]** |
| Other analyses | 17 | Report other analyses done—eg analyses of subgroups and interactions, and sensitivity analyses [**N/A]** |
| Discussion | | |
| Key results | 18 | Summarise key results with reference to study objectives [**line 395 to 396, line 423 to 446]** |
| Limitations | 19 | Discuss limitations of the study, taking into account sources of potential bias or imprecision. Discuss both direction and magnitude of any potential bias [**line 462 to 488]** |
| Interpretation | 20 | Give a cautious overall interpretation of results considering objectives, limitations, multiplicity of analyses, results from similar studies, and other relevant evidence |
| Generalisability | 21 | Discuss the generalisability (external validity) of the study results [**line 397 to 411, line 490 to 531]** |
| Other information | | |
| Funding | 22 | Give the source of funding and the role of the funders for the present study and, if applicable, for the original study on which the present article is based [**n/a]** |

*Give information separately for exposed and unexposed groups.

**Note:** An Explanation and Elaboration article discusses each checklist item and gives methodological background and published examples of transparent reporting. The STROBE checklist is best used in conjunction with this article (freely available on the Web sites of PLoS Medicine at http://www.plosmedicine.org/, Annals of Internal Medicine at http://www.annals.org/, and Epidemiology at http://www.epidem.com/). Information on the STROBE Initiative is available at www.strobe-statement.org.
